# The relationship between the number of chronic conditions and depressive symptoms in older adults with multimorbidity: the chain mediating role of psychological resilience and sleep quality

**DOI:** 10.3389/fpubh.2026.1973096

**Published:** 2026-10-22

**Authors:** Mengyuan Xu, Yingxin Duan, Zhiqiang Feng, Haoqi Li, Xiuming Sun, Jianing Yue, Jinkun Wang, Xianglan Zhuge, Xianqi Zhang, Shanju Hu, Fangfang Chen, Zhongming Chen

**Affiliations:** 1 School of Management, Shandong Second Medical University, Weifang, Shandong, China; 2 Health Management Research Center of Shandong Second Medical University, Weifang, Shandong, China; 3 School of Public Health, Shandong Second Medical University, Weifang, Shandong, China; 4 Affiliated Hospital of Shandong Second Medical University, Weifang, Shandong, China

**Keywords:** chronic disease management, depressive symptoms, older adults, psychological resilience, sleep quality

## Abstract

**Introduction:**

Depression represents a significant global public health challenge for older adults. The relationship between multimorbidity and depressive symptoms has been frequently studied. However, how the number of chronic conditions affects depressive symptoms in older adults with multimorbidity remains unclear. This study aimed to examine the relationship between the number of chronic conditions and depressive symptoms in older adults with multimorbidity, and to investigate the mediating roles of psychological resilience and sleep quality in this relationship.

**Methods:**

This study utilized cross-sectional survey data collected from two Chinese provinces between October 2024 and January 2025, involving 1,759 older adults with multimorbidity. Statistical analysis was performed using SPSS 21.0, with Spearman correlation analysis employed to explore associations between key variables. Furthermore, linear regression analysis using Model 6 in the PROCESS 4.2 macro was conducted to assess the relationship between the number of chronic conditions and depressive symptoms and to examine the chain-mediating effects of psychological resilience and sleep quality in this relationship.

**Results:**

Among older adults with multimorbidity, the number of chronic conditions was significantly associated with depressive symptoms (*β* = 0.094, 95% CI: 0.059, 0.130). Psychological resilience (*β* = 0.064, 95% CI: 0.042, 0.086) and sleep quality (*β* = 0.044, 95% CI: 0.030, 0.060) each mediated the relationship between the number of chronic conditions and depressive symptoms, accounting for 30.05 and 20.66% of the total effect, respectively. Additionally, a chain-mediating effect involving both psychological resilience and sleep quality was observed (*β* = 0.011, 95% CI: 0.007, 0.015), contributing 5.16% to the total effect.

**Conclusion:**

This study found that psychological resilience and sleep quality mediated the association between the number of chronic conditions and depressive symptoms in older adults with multimorbidity. In health management practice, greater attention could be given to the assessment of psychological resilience and sleep quality, together with psychological support and sleep health management, to support the comprehensive management of depressive symptoms. Due to the cross-sectional design, causal direction and temporal ordering cannot be inferred.

## 1 Introduction

Population aging has become a critical challenge for healthcare systems worldwide (1). Projections indicate that by 2050, China’s older adult population will increase substantially, with the population aged 65 and over reaching 400 million and those aged 80 and over reaching 150 million (2). With rapid population aging, depression among older adults has emerged as a major public health issue. The World Health Organization predicts that depression will become the leading cause of disease burden by 2030 (3). Existing research indicates that over 35% of older adults in China exhibit signs of depression (4). Depression in older adults not only diminishes quality of life but also increases morbidity, mortality, and healthcare costs (5). Multiple studies confirm that a combination of genetic, sociodemographic, psychosocial, lifestyle, and physiological factors collectively influence depression in older adults (6–13), with chronic disease identified as a significant risk factor (14).

Chronic non-communicable diseases, hereafter referred to as chronic diseases, do not refer to a single specific disease but rather to a group of conditions characterized by insidious onset, long duration, prolonged or persistent course, no clearly identified transmissible biological cause, and complex or uncertain etiology (15). Globally, about one-third of individuals with chronic diseases develop multimorbidity, defined as the co-occurrence of two or more chronic conditions (16, 17). In China, the proportion of adults aged 60 and over with two or more chronic diseases is as high as 76.5% (18). Individuals with multimorbidity require long-term treatment, care, and rehabilitation, imposing substantial financial pressure on families (19). Concurrently, multimorbidity is associated with high mortality, presenting a significant challenge to global healthcare systems (20). Existing research indicates that chronic diseases not only severely impact the physical health of older adults but also adversely affect their mental health (21). A growing body of epidemiological evidence finds a positive correlation between multimorbidity and depressive symptoms across various populations globally (22). A longitudinal cohort study based on middle-aged and older Chinese adults found that individuals with multimorbidity had a higher risk of depressive symptoms compared to those without chronic diseases (23). A meta-analysis encompassing over 380,000 participants confirmed that, compared to individuals with no chronic conditions or only one chronic condition, those with multimorbidity face a greater risk of experiencing depression (24).

Psychological resilience is defined as an individual’s ability to adapt to and recover from adversity, stress, and difficult life events (25). During the aging process, older adults may encounter adverse or stressful life events more frequently, including severe illness, retirement, loss of loved ones, marital conflict, and changes in social networks (26, 27). A substantial body of research has consistently demonstrated that chronic disease burden is associated with reduced psychological resilience, and that older adults with multiple chronic conditions tend to have lower levels of psychological resilience (28–30). Extensive empirical evidence across diverse populations has demonstrated that psychological resilience is negatively associated with depressive symptoms. Individuals with lower levels of psychological resilience tend to experience more severe depressive symptoms and greater difficulty in effectively adapting to stressful events (31–34).

Sleep is involved in regulating the body’s immune and metabolic processes. Sleep quality is crucial for the health and well-being of older adults (35), yet sleep problems are prevalent among older adults in various countries (36). Current studies show that individuals with chronic diseases often experience poor sleep quality due to compromised physiological function (37). In older adults, the presence of chronic diseases is associated with diminished sleep quality, and a higher chronic disease burden is associated with progressively worse sleep quality (38–41). Existing research indicates a strong association between poor sleep quality and depressive symptoms. Poorer sleep quality is associated with higher levels of psychological distress and depressive symptoms, a finding that has been consistently validated across multiple studies involving older adult populations in China, South Korea, and Brazil (42–44). Furthermore, existing research indicates a significant association between psychological resilience and sleep quality (45). Multiple studies have consistently demonstrated that psychological resilience is positively associated with sleep quality. Conversely, lower levels of psychological resilience are associated with greater worry, insomnia, and poorer sleep quality (46–48). Based on the hyperarousal theory of insomnia, lower levels of psychological resilience are associated with higher levels of pre-sleep cognitive hyperarousal, which in turn is associated with poorer sleep quality (49, 50). From the perspective of psychological resources, older adults with lower psychological resilience tend to have fewer psychological resources and are more likely to experience persistent discomfort, dyspnea, and other physical or psychological problems, which are associated with sleep quality impairments such as insomnia and frequent nocturnal awakenings (51). Individuals with lower psychological resilience are more prone to rumination, and pre-sleep rumination is associated with increased levels of cognitive arousal, which further interferes with sleep (52).

Existing research has explored the interrelationships among chronic disease, psychological resilience, sleep quality, and depressive symptoms; however, the specific roles of psychological resilience and sleep quality in these relationships remain unclear. The present study used the number of chronic conditions as the independent variable, and psychological resilience and sleep quality as mediating variables, to explore the associations between multimorbidity and depressive symptoms. This path analysis may help elucidate the underlying psychological mechanisms associated with the mental health of older adults with multimorbidity, thereby providing a theoretical basis for the development of targeted interventions. In summary, the present study proposed the following four hypotheses among older adults with multimorbidity: (1) there is a positive association between the number of chronic conditions and depressive symptoms; (2) psychological resilience mediates the association between the number of chronic conditions and depressive symptoms; (3) sleep quality mediates the association between the number of chronic conditions and depressive symptoms; and (4) psychological resilience and sleep quality play a chain mediating role in the association between the number of chronic conditions and depressive symptoms.

## 2 Methods

### 2.1 Participants

This study employed a multi-stage stratified random sampling method. First, two provinces, HN and HB, were selected as representative provinces based on their levels of economic development. In each province, one prefecture-level city with an economic development level at the provincial median was selected, namely hb and hs, respectively. Subsequently, based on high, medium, and low levels of socioeconomic development, three counties (districts) were randomly selected from each prefecture-level city. Within each county (district), four townships were further selected as sample townships. Finally, within each sample township, six villages were randomly selected, and 20–25 patients with chronic diseases were randomly chosen from each village for questionnaire surveys.

A total of 3,233 questionnaires were distributed in this survey, and 3,216 valid questionnaires were returned, yielding an effective response rate of 99.5%. Based on the study’s inclusion and exclusion criteria, a total of 1,759 samples were included. Inclusion criteria were: (1) aged 60 years or older; (2) diagnosed with at least two chronic disease; (3) all participants were informed and voluntarily agreed to participate. Exclusion criteria were: (1) patients with severe hearing or intellectual impairment; (2) participants with missing data. (3) Participants with a diagnosed mental disorder. Among the excluded participants, 17 cases (0.53% of the validly returned questionnaires) were excluded due to missing data. Given that the proportion of missing data was less than 1% and the missingness appeared to be randomly distributed, the risk of selection bias was relatively low, and the impact on sample representativeness and the reliability of the study findings was limited (Figure 1).

### 2.2 Measures

#### 2.2.1 Depressive symptoms

Depressive symptoms over the past week were assessed using the Center for Epidemiologic Studies Depression Scale (CES-D-10) (53). The scale was developed by Radloff at the National Institute of Mental Health in 1977 and consists of 10 items (54). Responses are scored on a 4-point scale from 0 to 3, with items 5 and 8 reverse-scored. The total score ranges from 0 to 30, with higher scores indicating more severe depressive symptoms. Participants with a score of ≥10 were considered to have depressive symptoms (55). This scale has been widely used and validated in studies involving Chinese older adults, demonstrating good reliability and validity (56, 57). In this study, the Cronbach’s α coefficient of this scale was 0.865.

#### 2.2.2 Number of chronic conditions

Multimorbidity was defined as the simultaneous presence of two or more chronic diseases. The study questionnaire included 12 categories of chronic diseases: hypertension, diabetes, dyslipidemia (hyperlipidemia or hypolipidemia), urinary system diseases (e.g., nephritis, renal failure), arthritis or rheumatism, heart disease (e.g., myocardial infarction, coronary heart disease, angina pectoris), chronic lung disease (e.g., chronic bronchitis, emphysema), cervical spondylosis, malignant tumors, mental illness (e.g., psychosis, depression), cerebral infarction, and digestive system diseases (e.g., chronic gastritis, gastric ulcer, cholecystitis). In this study, the number of chronic conditions in older adults was used to reflect their multimorbidity burden.

#### 2.2.3 Sleep quality

Sleep quality over the past month was assessed using the Pittsburgh Sleep Quality Index (PSQI). This scale was developed by Buysse et al. (58) and consists of seven components: (1) subjective sleep quality; (2) sleep latency; (3) sleep duration; (4) sleep efficiency; (5) sleep disturbances; (6) use of sleep medication; and (7) daytime dysfunction. Each component is scored from 0 to 3, yielding a global score ranging from 0 to 21. Higher scores indicate poorer sleep quality (59). This scale has been extensively used and validated in research involving Chinese older adults, showing good reliability and validity (60, 61). In this study, the Cronbach’s α coefficient of this scale was 0.745.

#### 2.2.4 Psychological resilience

Psychological resilience was measured using a five-item scale derived from the Chinese Longitudinal Healthy Longevity Survey (CLHLS) (62). The scale consists of the following five items: (1) No matter what happens, can you take things easy? (2) Do you feel that as you get older, you become less capable and have difficulty doing anything? (3) Do you often feel lonely and isolated? (4) Do you often feel nervous, frightened, or anxious? (5) Do you have the final say over your own affairs? Responses range from 1 to 5 (1 = always; 2 = often; 3 = sometimes; 4 = seldom; 5 = never). Items 1 and 5 were reverse-scored. The scores for all items were summed to obtain a total resilience score ranging from 5 to 25, reflecting participants’ current psychological adaptation characteristics, with higher scores indicating greater psychological resilience. These items have been validated in previous studies involving older adults, demonstrating good reliability and validity (63, 64). In this study, the Cronbach’s α coefficient of this scale was 0.703.

#### 2.2.5 Covariates

Based on previous research findings (65–67), the following control variables were included in this study: age (60–64, 65–69, 70–74, and ≥75 years), gender (male, female), marital status (unmarried, married, divorced, widowed), education level (primary school or below, junior high school, high school or vocational school, college or above), smoking status (smoker, non-smoker), alcohol consumption (drinker, non-drinker), health insurance participation (insured, not insured), and pension insurance participation (insured, not insured).

### 2.3 Statistical analysis

SPSS 21.0 was used for database construction, data management, and statistical analyses. First, descriptive statistical analyses were conducted to summarize the basic characteristics of the study participants. Second, Spearman correlation analysis was used to examine the correlations among the number of chronic conditions, psychological resilience, sleep quality, and depressive symptoms. Finally, mediation analysis was conducted using Model 6 of the PROCESS macro version 4.2 for SPSS (68), with the number of chronic conditions as the independent variable, psychological resilience and sleep quality as mediating variables, and depressive symptoms as the dependent variable. The bootstrap method with 5,000 resamples was used to estimate 95% confidence intervals, and an effect was considered statistically significant if the confidence interval did not include zero. In addition, considering the hierarchical clustering structure of the study participants, generalized estimating equations (GEE) were further used for sensitivity analysis. Township was specified as the clustering unit, with an independent working correlation structure and robust standard errors, to re-estimate the major regression pathways and assess the robustness of the study findings. All regression coefficients reported in this study were standardized coefficients (*β*), and all continuous variables were standardized before being entered into the models.

## 3 Results

### 3.1 Basic characteristics of participants

The basic characteristics of the participants are shown in Table 1. The study ultimately included 1,759 older adults with multimorbidity, with 669 (38.03%) aged 75 years and above and 1,150 (65.38%) being female. The majority of older adults were married, accounting for 1,241 (70.55%). The highest number had a primary school education or below, accounting for 1,195 (67.94%). Most older adults did not smoke or drink alcohol, with 1,511 (85.90%) and 1,549 (88.06%), respectively. Health insurance and pension insurance coverage were very high, at 1,748 (99.37%) and 1,725 (98.07%), respectively. Participants with 2 chronic diseases were the most common, accounting for 884 individuals (50.26%), while those with 4 or more chronic diseases accounted for 333 individuals (18.93%). The results of this study showed that there were significant differences in the levels of depressive symptoms among groups with different demographic characteristics (*p* < 0.05). Specifically, older adults who were of advanced age, female, widowed, had low educational levels, or had a higher number of chronic diseases experienced more severe depressive symptoms.

**Table 1:** Basic characteristics of participants.

Variable	Total sample [*n* (%)]	No depressive symptoms [*n* (%)]	Depressive symptoms [*n* (%)]	*χ^2^*	*P*
Age				13.328	0.004
60–64	241 (13.70)	150 (16.11)	91 (10.99)		
65–69	313 (17.79)	172 (18.47)	141 (17.03)		
70–74	536 (30.47)	282 (30.29)	254 (30.68)		
≥75	669 (38.03)	327 (35.12)	342 (41.30)		
Gender				3.900	0.048
Male	609 (34.62)	342 (36.73)	267 (32.25)		
Female	1,150 (65.38)	589 (63.27)	561 (67.75)		
Marital Status				22.628	<0.001
Unmarried	18 (1.02)	15 (1.61)	3 (0.36)		
Married	1,241 (70.55)	690 (74.11)	551 (66.55)		
Divorced	7 (0.40)	4 (0.43)	3 (0.36)		
Widowed	493 (28.03)	222 (23.85)	271 (32.73)		
Education Level				18.493	<0.001
Primary or below	1,195 (67.94)	597 (64.12)	598 (72.22)		
Junior High School	417 (23.71)	235 (25.24)	182 (21.98)		
High School/Vocational	137 (7.79)	92 (9.88)	45 (5.43)		
College or above	10 (0.57)	7 (0.75)	3 (0.36)		
Smoking Status				0.200	0.654
No	1,511 (85.90)	803 (86.25)	708 (85.51)		
Yes	248 (14.10)	128 (13.75)	120 (14.49)		
Alcohol Drinking				0.322	0.570
No	1,549 (88.06)	816 (87.65)	733 (88.53)		
Yes	210 (11.94)	115 (12.35)	95 (11.47)		
Health Insurance				2.924	0.087
No	11 (0.63)	3 (0.32)	8 (0.97)		
Yes	1748 (99.37)	928 (99.68)	820 (99.03)		
Pension Insurance				3.004	0.083
No	34 (1.93)	13 (1.40)	21 (2.54)		
Yes	1725 (98.07)	918 (98.60)	807 (97.46)		
Number of chronic diseases					
2	884 (50.26)	537 (57.68)	347 (41.91)	57.734	<0.001
3	542 (30.81)	271 (29.11)	271 (32.73)		
≥4	333 (18.93)	123 (13.21)	210 (25.36)		

**Table 2:** Descriptive statistics and correlation analysis.

Variable	Mean ± SD	Number of chronic conditions	Psychological resilience	Sleep quality	Depressive symptoms
Number of chronic conditions	2.794 ± 1.006	1			
Psychological resilience	18.832 ± 3.476	−0.151***	1		
Sleep quality	7.872 ± 4.428	0.179***	−0.295***	1	
Depressive symptoms	9.800 ± 6.536	0.211***	−0.564***	0.478***	1

***p < 0.001, **p < 0.01, *p < 0.05.

### 3.2 Descriptive statistics and correlation analysis

The mean score for the number of chronic conditions was 2.794 ± 1.006, for depressive symptoms was 9.800 ± 6.536, for psychological resilience was 18.832 ± 3.476, and for sleep quality was 7.872 ± 4.428. Spearman correlation analysis showed that the number of chronic conditions was negatively correlated with psychological resilience (*r* = −0.151, *p* < 0.001) and positively correlated with poorer sleep quality (*r* = 0.179, *p* < 0.001) and depressive symptoms (*r* = 0.211, *p* < 0.001). Psychological resilience was negatively correlated with depressive symptoms (*r* = −0.564, *p* < 0.001) and poorer sleep quality (*r* = −0.295, *p* < 0.001). Poorer sleep quality was positively correlated with depressive symptoms (*r* = 0.478, *p* < 0.001). Significant pairwise correlations were observed among all core variables (Table 2).

### 3.3 Multiple mediation analysis results of psychological resilience and sleep quality between number of chronic conditions and depressive symptoms

After controlling for confounding factors, the regression results of the multiple mediation model are shown in Table 3. The number of chronic conditions was significantly associated with lower psychological resilience (*β* = −0.131, *P* < 0.001), poorer sleep quality (*β* = 0.138, *P* < 0.001), and higher levels of depressive symptoms (*β* = 0.094, *P* < 0.001). Psychological resilience was significantly negatively associated with poorer sleep quality (*β* = −0.255, *P* < 0.001) and depressive symptoms (*β* = −0.483, *P* < 0.001), while poorer sleep quality was significantly positively associated with depressive symptoms (*β* = 0.317, *P* < 0.001).

**Table 3:** Results of the mediation effect model.

Variable	Psychological resilience		Sleep quality		Depressive symptoms	
	*β*	*t*	*β*	*t*	*β*	*t*
Number of chronic conditions	−0.131	−5.594***	0.138	6.139***	0.094	5.210***
Psychological resilience			−0.255	−11.241***	−0.483	−25.795***
Sleep quality					0.317	16.662***
Age (reference: 60–64 years)						
65–69 years	−0.007	−0.218	0.021	0.671	0.009	0.356
70–74 years	−0.052	−1.413	−0.007	−0.208	0.006	0.227
≥75 years	−0.040	−1.051	0.009	0.249	0.015	0.516
Gender (reference: male)						
Female	−0.023	−0.783	0.148	5.418***	0.009	0.410
Marital Status (reference: married)						
Non-married ^a^	−0.089	−3.656***	0.019	0.833	0.036	1.966*
Education Level (reference: primary school or below)						
Junior high school	0.082	3.252**	−0.033	−1.372	0.020	1.033
High school and above	0.036	1.452	−0.083	−3.491***	−0.011	−0.587
Smoking Status (reference: yes)						
No	−0.035	−1.273	−0.003	−0.121	−0.057	−2.739**
Alcohol Drinking (reference: yes)						
No	−0.038	−1.417	−0.020	−0.777	−0.003	−0.153
Health Insurance (reference: yes)						
No	0.001	0.029	−0.016	−0.693	0.035	1.902
Pension Insurance (reference: yes)						
No	−0.055	−2.254*	0.076	3.258**	−0.016	−0.845
*R*	0.237		0.389		0.680	
*R^2^*	0.056		0.151		0.463	
*F*	8.617***		23.882***		107.257***	

***p < 0.001, **p < 0.01, *p < 0.05. The coefficients reported in the table are standardized regression coefficients (β). ^a^Non-married includes single, divorced, or widowed.

**Figure 1:**
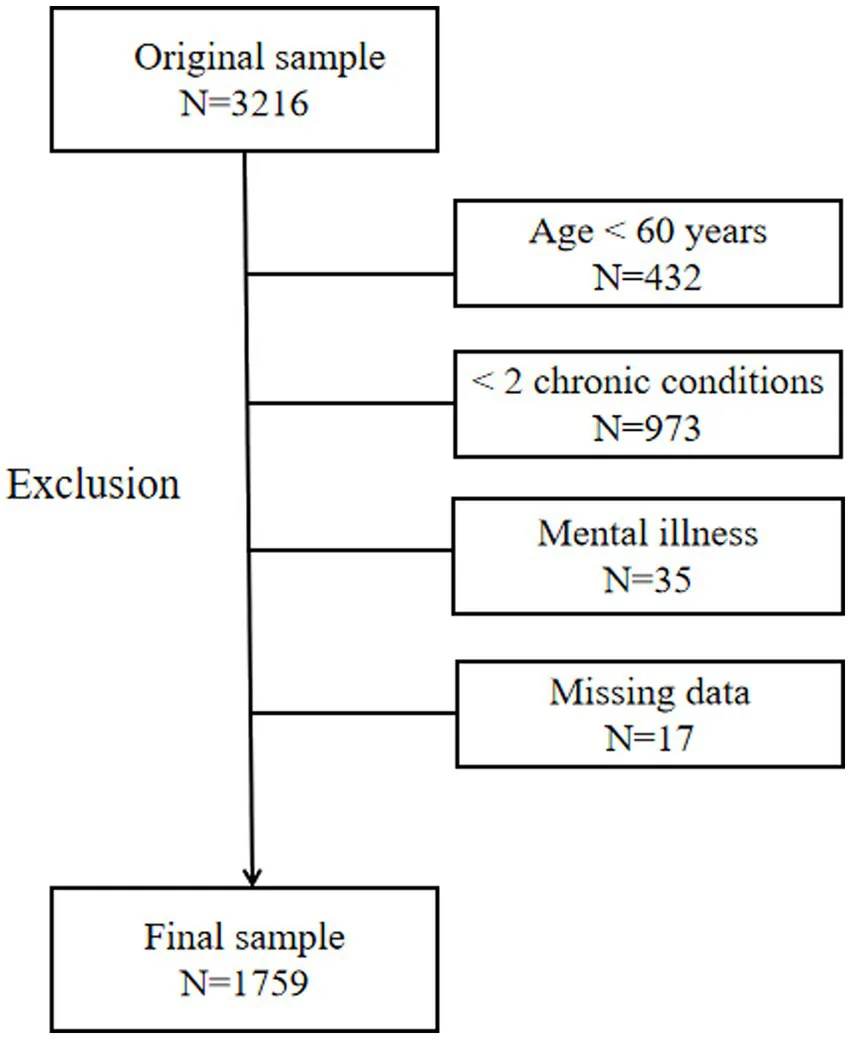
Flowchart of participant selection.

The bias-corrected percentile bootstrap method (with 5,000 resamples) was used to test the mediating effects. Table 4 shows the mediating effect values of psychological resilience and sleep quality in the relationship between the number of chronic conditions and depressive symptoms. The direct effect of the number of chronic conditions on depressive symptoms was 0.095, accounting for 44.60% of the total effect. The total indirect effect of psychological resilience and sleep quality between the number of chronic conditions and depressive symptoms was 0.118. The first path was: Number of Chronic Conditions → Psychological Resilience → Depressive Symptoms (*β* = 0.064; 95% CI: 0.042, 0.086). The second path was: Number of Chronic Conditions → Sleep Quality → Depressive Symptoms (*β* = 0.044; 95% CI: 0.030, 0.060). The third path was: Number of Chronic Conditions → Psychological Resilience → Sleep Quality → Depressive Symptoms (*β* = 0.011; 95% CI: 0.007, 0.015). The proportions of these three indirect effects relative to the total effect were 30.05%, 20.66%, and 5.16%, respectively, and none of the confidence intervals included zero. The results showed that psychological resilience and sleep quality each exhibited significant independent mediating effects in the association between the number of chronic conditions and depressive symptoms, while the two variables also exhibited a significant chain mediating effect. The specific pathways are shown in Figure 2.

**Table 4:** Mediation effect analysis of psychological resilience and sleep quality.

Pathway	Effect	*SE*	95% CI		Mediation (%)
Total effect	0.213	0.023	0.167	0.259	100
Direct effects	0.095	0.018	0.059	0.130	44.60
Total indirect effect	0.118	0.015	0.089	0.148	55.40
Number of Chronic Conditions → Psychological resilience → Depressive symptoms	0.064	0.011	0.042	0.086	30.05
Number of Chronic Conditions → Sleep quality → Depressive symptoms	0.044	0.008	0.030	0.060	20.66
Number of Chronic Conditions → Psychological resilience → Sleep quality → Depressive symptoms	0.011	0.002	0.007	0.015	5.16

**Figure 2:**
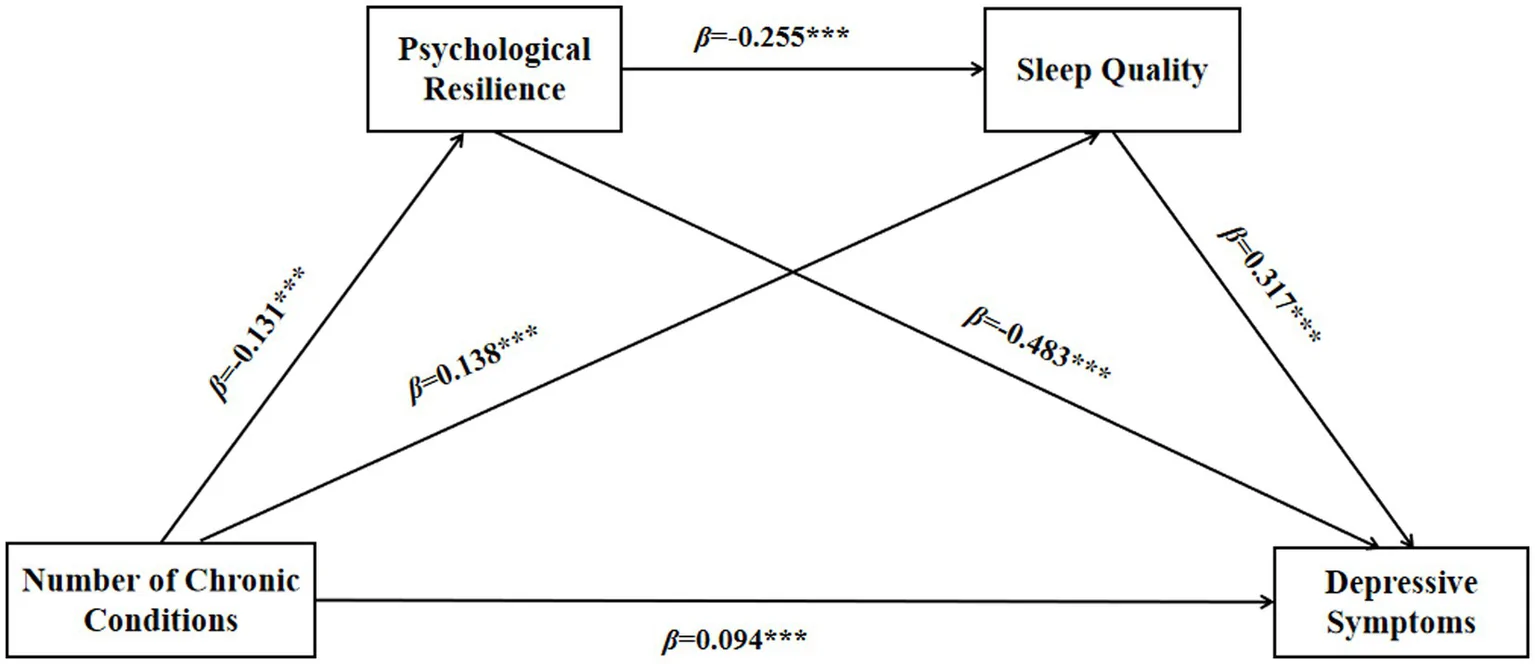
Path diagram of the chain mediation effect. ***p < 0.001, **p < 0.01,*p < 0.05.

To examine a possible alternative direction between sleep quality and depressive symptoms, we further tested the alternative pathway “number of chronic conditions → depressive symptoms → sleep quality.” The results showed that this indirect pathway was also statistically significant, indicating that the cross-sectional data cannot exclude the possibility that depressive symptoms may precede changes in sleep quality. Therefore, the current data are insufficient to determine the temporal ordering among these variables.

### 3.4 Sensitivity analysis

To further account for the hierarchical clustering structure of the sample, we re-estimated the major regression pathways using generalized estimating equations (GEE), with township specified as the clustering unit and robust standard errors applied. The results showed that the number of chronic conditions was significantly negatively associated with psychological resilience (*β* = −0.132; SE = 0.026; 95% CI: −0.183, −0.081; *P* < 0.001), and significantly positively associated with poorer sleep quality (*β* = 0.138; SE = 0.023; 95% CI: 0.094, 0.182; *P* < 0.001) and depressive symptoms (*β* = 0.095; SE = 0.022; 95% CI: 0.052, 0.137; *P* < 0.001). Psychological resilience was significantly negatively associated with poorer sleep quality (*β* = −0.254; SE = 0.028; 95% CI: −0.308, −0.200; *P* < 0.001) and depressive symptoms (*β* = −0.482; SE = 0.027; 95% CI: −0.535, −0.429; *P* < 0.001), while poorer sleep quality was significantly positively associated with depressive symptoms (*β* = 0.319; SE = 0.025; 95% CI: 0.271, 0.367; *P* < 0.001).

## 4 Discussion

Depression poses a challenge in geriatric health management. In this study of 1,759 older adults, data revealed that the severity of depressive symptoms was more prominent among those who were of advanced age, female, widowed, had lower educational attainment, or presented with a greater number of chronic conditions. Advanced age is accompanied by physiological decline and diminished social roles, which, together with the symptom burden of multimorbidity, may be associated with increased feelings of helplessness and loss of value (69). Women may have greater emotional sensitivity and more nuanced emotional experiences and may therefore experience stronger psychological stress responses when facing negative life events (70). Widowhood, as a major negative life event, not only causes emotional loss but also often weakens practical support systems, exacerbating loneliness and the risk of depression (71). Lower education levels may also limit health literacy, psychological adjustment strategies, and access to social resources, making it more difficult to cope effectively with the psychological stress associated with illness (72). The number of chronic conditions was positively correlated with the severity of depressive symptoms, a result consistent with previous studies, thereby supporting Hypothesis 1 (73, 74). From a biological perspective, chronic conditions are often accompanied by elevated levels of systemic inflammation (75, 76) and may accelerate aging-related neuropathological changes, such as gray matter atrophy (77, 78), both of which are associated with depression risk. From a psychosocial perspective, on the one hand, the persistent economic pressure associated with multiple chronic conditions may create a vicious cycle extending from the individual to the entire family (79). On the other hand, patients, particularly older adults, may be compelled to reduce basic living expenditures on nutrition and social activities because of the burden of multiple medications, which may be associated with poorer quality of life and reduced feelings of future security (80). The combined burden of economic hardship and life deprivation is associated with higher levels of psychological distress and depressive symptoms.

Among older adult patients with multimorbidity, psychological resilience plays an indirect role in the association between the number of chronic conditions and depressive symptoms, supporting Hypothesis 2. A higher chronic disease burden is significantly associated with lower levels of psychological resilience (81). From a physiological perspective, multimorbidity triggers inflammatory responses and oxidative stress, which damage the nervous system and impair HPA axis function. These physiological changes are associated with lower levels of psychological resilience (82). From a socio-psychological perspective, older adults with multimorbidity are required to continuously adapt to physiological, psychological, and social declines. This process is associated with a decrease in self-confidence and sense of control, which may affect their ability to effectively mobilize sufficient psychosocial resources to maintain mental health and balance. These factors may be associated with lower levels of psychological resilience (83). Specifically, older adults with multimorbidity often face negative events such as treatment burden, disease symptoms, unsatisfactory treatment outcomes, and communication barriers with healthcare professionals (84, 85). These negative events, as stressors, may adversely affect the patient’s psychological state and mood, and are further associated with lower levels of psychological resilience (86). Existing research shows that psychological resilience is significantly associated with depressive symptoms among older adults with multimorbidity (87, 88). From a physiological perspective, individuals with low psychological resilience often exhibit significantly insufficient adaptive regulatory capacity in the brain’s reward and emotion regulation systems when facing chronic stress, which may be associated with more severe depressive symptoms (89). From a social-psychological perspective, on one hand, chronic disease patients with lower resilience often struggle to cope positively and optimistically with various difficulties in treatment or daily life, such as pain, fatigue, and medication burden. This may be closely associated with negative emotional states such as depression (90). On the other hand, older adults with lower resilience often tend to adopt a negative attributional style, display pessimistic attitudes toward life, and are unable to effectively cope with stressful events (91), which is associated with more severe depressive symptoms (92).

Our study also demonstrates that, in older adults with multimorbidity, sleep quality plays an indirect role in the association between the number of chronic conditions and depressive symptoms, thereby supporting Hypothesis 3. Multimorbidity is significantly positively correlated with the incidence of sleep disorders (93). First, chronic conditions are often accompanied by long-term pain and discomfort, and pain is closely associated with sleep disturbances. Second, multimorbidity is closely related to declining physical function, disrupted sleep architecture, and circadian rhythm disturbances, and this association is particularly prominent in older adults (94). Furthermore, managing multimorbidity often involves polypharmacy, and the use of multiple medications is associated with sleep disturbances. For example, bronchodilators, beta-blockers, central nervous system stimulants, gastrointestinal drugs, and some cardiovascular medications have been reported to be associated with sleep problems (95). Regarding the relationship between sleep quality and depressive symptoms, existing research indicates that sleep problems may adversely affect mood and psychological well-being and are associated with higher levels of depressive symptoms (96). From a physiological perspective, long-term poor sleep quality, insufficient sleep duration, and sleep disruption may contribute to neurochemical imbalances and impaired emotion regulation, which may be related to the onset or worsening of depressive symptoms (97). Clinical observations also indicate a higher incidence of depression among older adults using hypnotic medications (benzodiazepines), suggesting a close relationship between sleep and depression (98). Furthermore, the sense of loss of control induced by insomnia may activate learned helplessness, which is associated with the development of depression (99). This study further confirms a significant association between psychological resilience and sleep quality. According to the hyperarousal hypothesis of insomnia, difficulties in falling asleep or maintaining sleep are closely associated with increased cognitive arousal and emotional dysregulation. Individuals with lower levels of psychological resilience often struggle to effectively regulate negative emotions and control pre-sleep cognitive arousal. This persistent state of cognitive and emotional activation may prolong sleep onset latency, increase the frequency of nocturnal awakenings, and be associated with poorer sleep quality (100). From a psychological perspective, lower levels of psychological resilience are closely related to difficulties in emotion regulation when facing chronic stress. Individuals with poorer psychological resilience tend to be more susceptible to excessive worry and rumination about disease consequences, treatment processes, and future uncertainties. This sustained state of cognitive and emotional arousal may interfere with normal sleep initiation and is associated with frequent nocturnal awakenings as well as decreased sleep quality (101, 102).

This study also revealed a significant pathway: Number of Chronic Conditions → Psychological Resilience → Sleep Quality → Depressive Symptoms. This model suggests that the chain relationship between psychological resilience and sleep quality plays a mediating role in the association between the number of chronic conditions and depressive symptoms. That is, among older adults with multimorbidity, a greater number of chronic conditions is associated with lower levels of psychological resilience, which in turn is associated with poorer sleep quality, and poorer sleep quality is further associated with higher levels of depressive symptoms (103–105). This model suggests that multimorbidity, as a persistent stressor, may be associated with depletion of psychological resilience and difficulty in effectively alleviating negative emotions, which is subsequently associated with poorer sleep quality, while worsening sleep quality may also be associated with an increased risk of depression. Notably, although the chain mediating effect was statistically significant, its effect size was relatively small, accounting for only 5.16% of the total effect. Therefore, this finding is more appropriately interpreted as one component of the complex association between chronic disease burden and depressive symptoms, rather than as evidence that this sequential pathway has a substantial practical intervention effect. Meanwhile, further alternative pathway analysis showed that the indirect association “Number of Chronic Conditions → Depressive Symptoms → Sleep Quality” was also statistically significant. This finding suggests that bidirectional associations may exist among these variables and further indicates that cross-sectional data cannot determine their actual temporal ordering.

This study has several limitations. First, the cross-sectional design only allows observation of variables at a single point in time, precluding the establishment of temporal ordering or causal relationships among the variables. For example, depressive symptoms may have reverse or bidirectional associations with lower psychological resilience and poorer sleep quality, and the temporal ordering between psychological resilience and sleep quality also cannot be determined. Future research could employ longitudinal tracking to dynamically observe variable changes and clarify causal mechanisms and dynamic bidirectional relationships. Second, the data rely on self-reports from older adults, which may be subject to recall bias affecting result reliability. In addition, some items in the psychological resilience scale are similar in content to certain depressive symptoms assessed by the CES-D-10. Third, this study mainly focused on the associative pathways involving psychological resilience and sleep quality between the number of chronic conditions and depressive symptoms, but other pathways may also exist. Fourth, this study employed a multistage stratified random sampling method. Although sensitivity analyses were conducted with township as the clustering unit, within-village correlation cannot be completely ruled out. Fifth, although some of the correlations between variables in this study reached statistical significance, the effect sizes were small. Finally, although the study controlled for several covariates such as age and gender, other unmeasured confounding factors may still influence the results.

## 5 Conclusion

This study extends the existing literature by revealing a significant association between the number of chronic conditions and depressive symptoms in older adults with multimorbidity. The results showed that, among older adults with multimorbidity, there is a complex set of associations among the number of chronic conditions, psychological resilience, sleep quality, and depressive symptoms. Psychological resilience and sleep quality may be important factors for understanding the association between chronic disease burden and depressive symptoms in older adults with multimorbidity. Therefore, in the health management of older adults with multimorbidity, greater attention could be given to the assessment of psychological resilience and sleep quality, together with measures such as psychological support, stress-coping guidance, sleep health education, and screening for sleep problems, to support the comprehensive management of depressive symptoms. Given the cross-sectional design of this study, the findings cannot be used to determine the temporal ordering or causal relationships among these variables. Future longitudinal studies are needed to further examine the dynamic relationships and potential pathways among the number of chronic conditions, psychological resilience, sleep quality, and depressive symptoms, thereby providing a basis for mental health management and related intervention research among older adults with multimorbidity.

## Data Availability

The raw data supporting the conclusions of this article will be made available by the authors, without undue reservation.
